# Water Soluble G-protein Coupled Receptor Enabled Biosensors

**DOI:** 10.31480/2330-4871/095

**Published:** 2019-08-13

**Authors:** Renyu Liu, A. T. Charlie Johnson, Jeffery G. Saven

**Affiliations:** 1 Department of Anesthesiology and Critical Care, Perelman School of Medicine, University of Pennsylvania, USA; 2 Department of Physics and Astronomy, University of Pennsylvania, USA; 3 Department of Chemistry, University of Pennsylvania, USA

## Abstract

This article will briefly overview our efforts in the engineering of water soluble variants of a G-protein coupled receptor (GPCR) and its novel applications to develop biosensors using such water soluble variants of GPCR. While the technologies using water soluble GPCR are still under development, they offer new tools and strategies to study the function of GPCR, explore potential new compounds for potential clinical usage, and monitor endogenous peptides in various physiological and pathological conditions.

This article will briefly overview our efforts in the engineering of water soluble variants of a G-protein coupled receptor (GPCR). GPCR is a category of proteins that govern many physiological and pathological processes in the human body. A GPCR consists of 7 helical domains spanning across a membrane. It can be activated with agonists and blocked with antagonists. It is estimated that about 35% of all the medications approved by the Food and Drug Administration (FDA) in the United States and by European regulatory agencies have modes of action that involve GPCRs. This represents over 700 medications targeting various diseases and medical conditions [1,2]. Many cutting edge technologies have been developed to study GPCRs to reveal their signaling pathways and to discover new medications for clinical use. Such approaches include high resolution structural analysis for binding site analysis and drug design, receptor engineering, cheminformatics, virtual screening, high-fidelity screening, and many other molecular pharmacology methodologies [3–6].

Many medications used in perioperative and pain medicine target GPCRs. These include beta-adrenergic receptors (epinephrine, a medication used in various situation to treat shock status, anaphylaxis, asthmatic attack etc.), dopamine receptors (dopamine, a medication to treat hypotension), vasopressin receptors (vasopressin, a medication to treat various hypotensive shock), and opioid receptors (morphine, a medication to treat severe acute and chronic pain) as well as many others. Opioid receptors are a group of receptors with high structural similarity to other GPCR family members. Despite the problems of addiction, diversion, and many serious side effects, the therapeutic use of opioids continues to play a major role in medicine, especially during the perioperative period for acute pain treatment and in pain medicine for chronic pain management [7–9]. As a result of this and other factors, the amount of opioid consumption has soared in the United States and many other countries [9–12].

Complications and adverse effects from opioid administration fall within two broad categories: non-life threatening (nausea, vomiting, pruritus, constipation, muscle rigidity, and cognitive impairment, etc) and life threatening (addiction and respiratory depression) [8,13,14]. Many challenges confront scientists, clinicians, lawmakers, and politicians to curb the epidemic of opioid related deaths and injuries [15]. It is thought that both the analgesic and side effects are transduced through the mu opioid receptor (MOR) [13,16]. Therefore, the MOR, a typical GPCR, has been a hot target for investigation. The information derived from this specific receptor can potentially be applicable to other GPCRs. Our multi-disciplinary team has been working on developing novel approaches to study ligand and receptor interactions with a specific focus on MOR [4,6,17–20].

Realizing the inherent difficulty of studying structure and function relationships of membrane bound proteins due to technilogical issues we developed variants of membrane proteins that include a water soluble MOR (wsMOR). These variants can be isolated in large quantities from *E. coli* over-expression systems and be suitable for a wide variety of high-resolution biophysical and drug discovery studies [6,19,21–23]. We initiated this work using homology modeling when the crystal structures of opioid receptors were not yet available, and then modified the variants with the best crystal structure information when it became available. Figure 1 indicates our work flow for the receptor engineering process of designing water soluble variants. The final product of the engineering process may result in one or more sequences for the variants. We simulate 3-dimensional structure models to assess stability in a water environment. The molecular dynamics simulation shown in Figure 2A, indicates that one of our engineered wsMOR-TM sequences remains stable in a water enviromonet after 75 ns simulation. The sequence is then encoded for protien expression in *E. coli* and then purified to demonstrate whether the product is suitable for ligand interaction explorations. Figure 2B shows our purification process for one of the variants we have designed. Based on the outcome of the expression and characterization, we may return to the engineering process to modify the mutations and obtain new vairiants to test new hypotheses. Figure 3 shows one of our variants, named as wsMOR-G, that we are characterizing in an attempt to demonstrate that the receptor can tolerate additional mutations on the surface of the protein. This variant can be over-expressed and purified from *E. coli*. Our prelimianary data indicates that its affinity (dissociation constant) with morphine is around K_D_ = 3.5 nM.

In an attempt to demonstrate that the approach we used to engineer wsMOR is expandable to other GPCRs, we are using adregergic receptor as another target for a feasibility test. We will report these results in other occasions. These efforts cleary indicated that our water solubilization approach can potentialy be used with other GPCRs for structure function studies.

## Graphene Enabled Biosensors Using wsMOR

**Figure 5:**
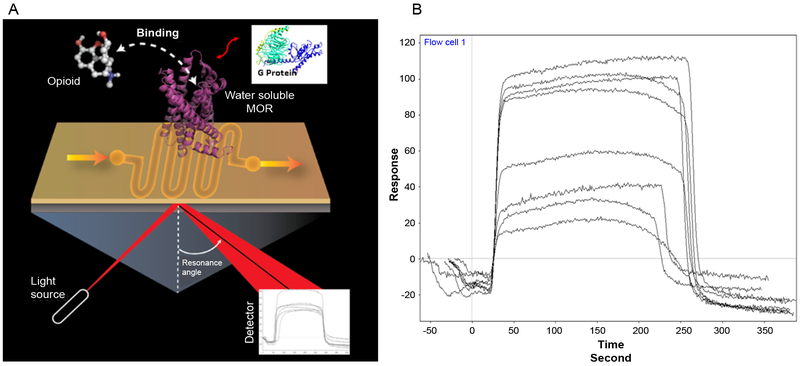
Illustration of the Surface Plasmon resonance enabled biosensor system for ligand and G protein interactions with mu opioid receptor (MOR). Water soluble MOR was immobilized on the surface of a sensor chip for the surface Plasmon resonance. A model opioid molecule and the G protein model were shown in the image for potential interactions (Figure 5A). Characteristic response can be detected with the detector as show in Figure 5B for a sample response output from various concentrations of morphine.

Since there are no membranes or membrane surrogates required, it is possible to use the wsMOR for direct opioid interaction and activation monitoring purposes using biosensor enabled technologies. Using these advances in obtaining functional forms of wsMOR, we were able to manipulate the receptor outside of a biomembrane and develope a novel, all-electronic biosensor for opioids. It consists of wsMOR chemically bonded to a graphene field-effect transistor to read out ligand binding as indicated in Figure 5.

A shadow mask process was developed to fabricate arrays of hundreds of graphene transistors with average mobility of ~1500 cm^2^ V^−1^ s^−1^ and yield exceeding 98%. We have demonstrated that the biosensor exhibits high sensitivity and selectivity for opioid ligands, including small molecules and large endorphin peptides with a detection limit of 10 pg/mL [18,19]. This opens the novel possibility of monitoring opioid and opioid interactions using electronic readouts in a real time manner. Theoretically it is also possible to monitor G protein interactions with the receptor using this system. We are devoting our effects to reveal how the receptor interacts with G proteins to elucidate factors that may affect G protein assocation and dissociation.

## Surface Plasma Resonance (SPR) Enabled Biosensors Using wsMOR

We are also developing a surface plasmon based sensor platform to immobolize the engineered wsMOR for the purpose of thermodynamic analsyis of opioid-MOR interactions. SPR enabled biosensors have been the classic method for investigating ligand protein interactions in a lipid and lable free system. However, immobilizing a membrane protein such as a GPCR, onto an SPR biosensor has been very challenging. Our wsMORs made immobolization of the receptor onto the sensor much simpler. Figure 5a demonstrates the key components of system. As long as the receptor is successfully immobolized on the sensorchip, the interacton of the ligand with the receptor can be detected for the purpose of calculating both association and dissociation constants. As noted in the figure, the interaction of the receptor with G protien can also be monitored. Such interactions can be explored in the presence or absence of opioid ligand. Figure 5b shows typical concentration dependent SPR signals of morphine interaction. Our preliminary data indicates the K_D_ for morphine for the above mentioned wsMOR-G is around 3 nM.

In conclusion, varaints of wsMOR offer valuable tools for exploring ligand and G protein interactions with the receptor in a lipid and labeling free system.

## Figures and Tables

**Figure 1:**
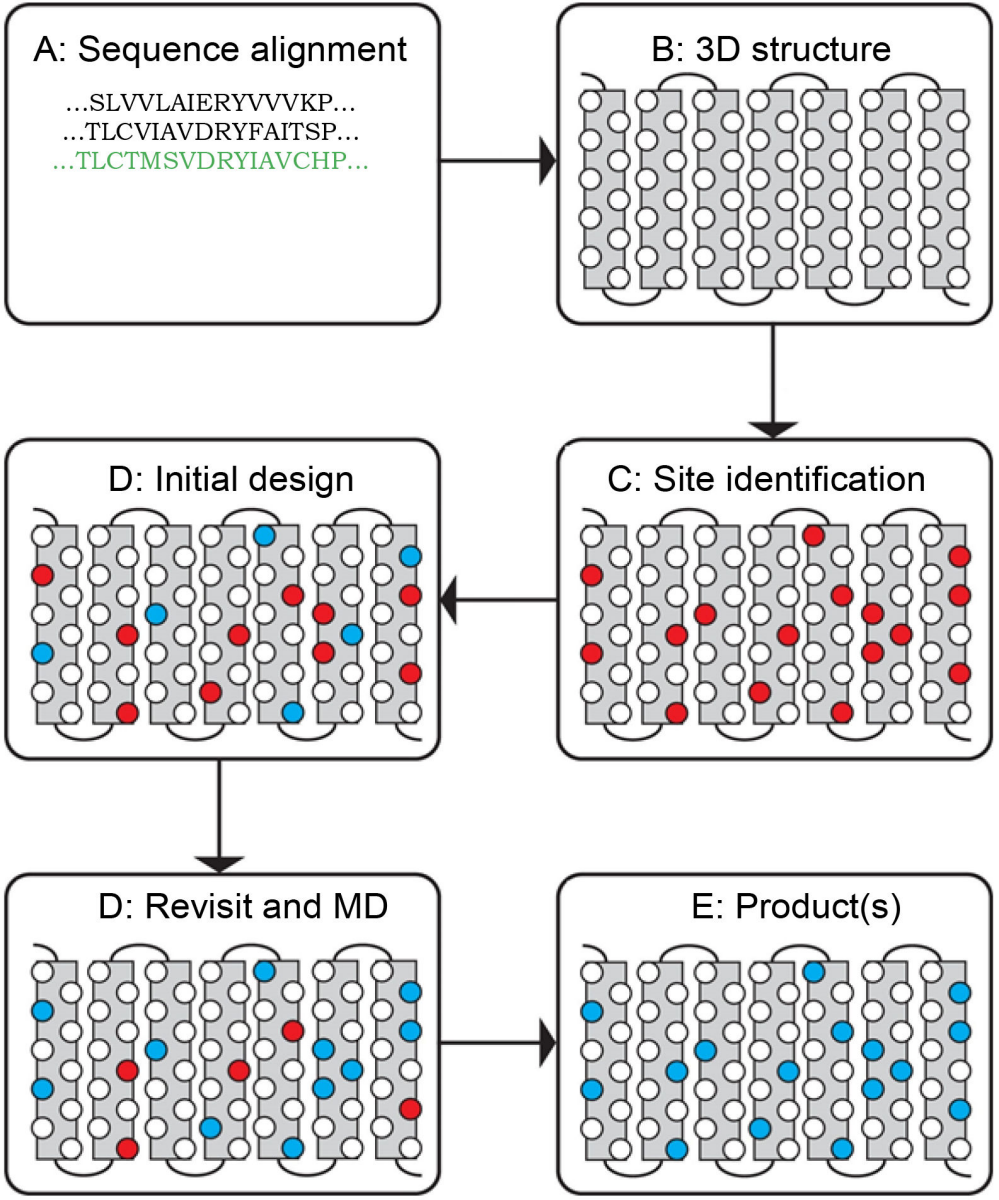
The work-flow of the water-soluble G-protein coupled receptor engineering process. From A for sequence alignment if there is no available crystal structure, to B for a homology modeling. If the crystal structure is readily available, the process could start from here to identify solvent exposed residues on the surface of the transmembrane domain. After the eligible hydrophobic residues are identified, we will move to the step D for the initial computational design, then move to structural analysis and molecular dynamics simulation (MD) to ensure the engineered protein could stay in aqua solution without significant disturbance of the structure. The product of the engineering will be one or a set of sequences that can be tested in expression system. Image courtesy of Prof. Jose Manuel Perez-Aguilar.

**Figure 2:**
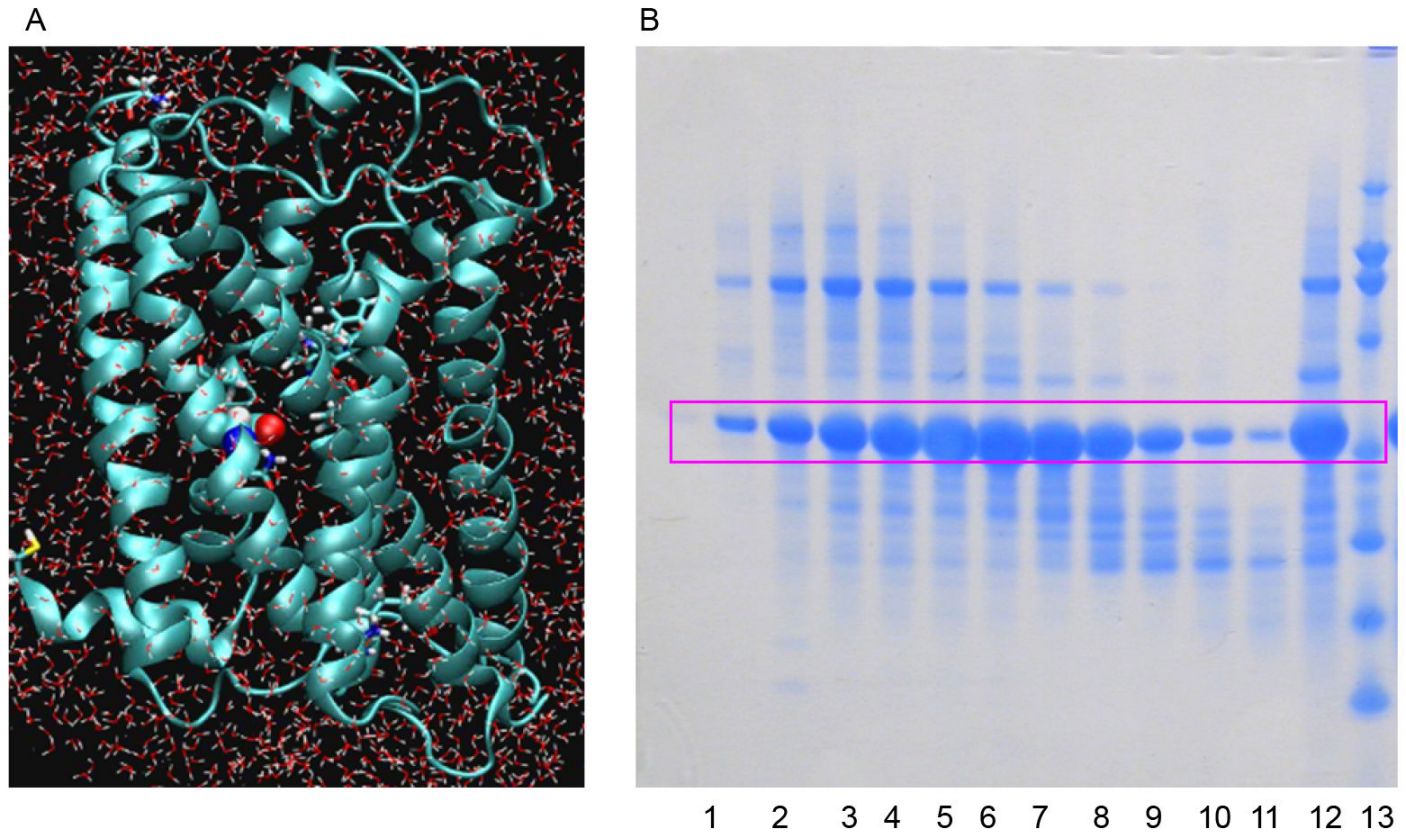
2A indicates one of our engineered water soluble variants opioid receptor undergoing molecular dynamics simulation evaluations to see whether it could remain structurally stable in a water environment. 2B, a Coomasie stained polyacrylamide electrophoresis gel, shows how one of the engineered variants of water soluble human mu opioid receptor can be expressed and purified. Lane 12 is the crude material from *E. Coli*, lanes 1–11 demonstrate various attempts at purifying the protein. Lane 13 is the marker of the gel.

**Figure 3:**
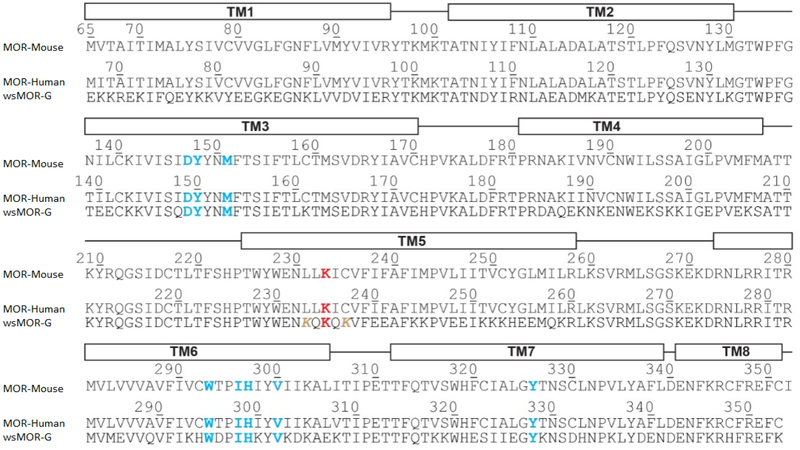
The sequence comparison of a water-soluble variant of human mu opioid receptor (MOR), named as wsMOR-G. with the sequences of native mouse and human MOR. Some of the well conserved residues among them are colored in blue or red. Image courtesy of Prof. Jose Manuel Perez-Aguilar.

**Figure 4:**
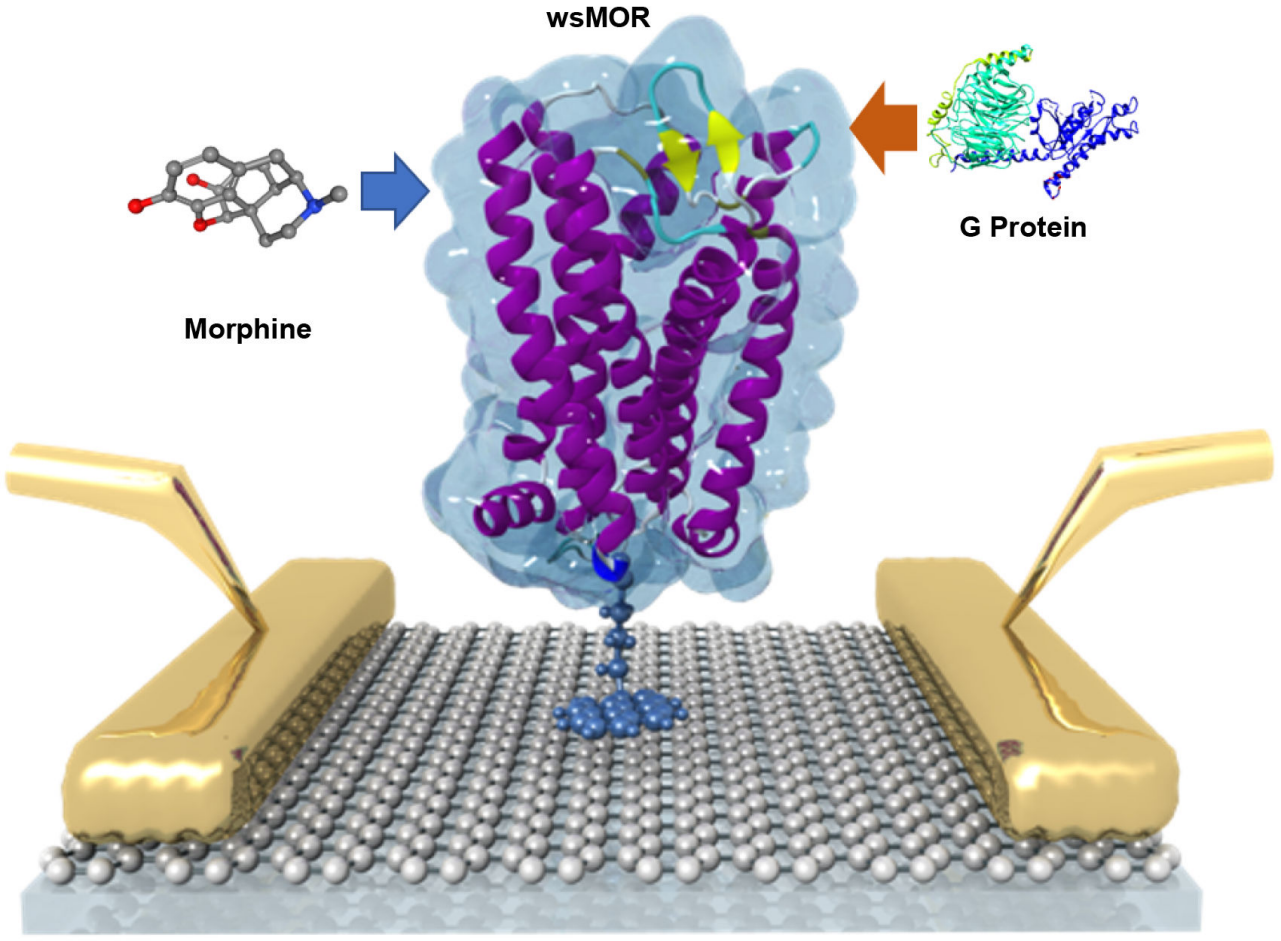
Illustration of the graphene enabled biosensor system for ligand and G protein interactions with mu opioid receptor (MOR). Morphine was shown as an example of MOR full agonist. Water soluble MOR (wsMOR) was immobilized on the surface of a thin layer of graphene, the faradaic current may change in the presence of the different concentrations of morphine or G protein. Based on the changes of the faradaic current changes, the affinity of morphine or the G protein with the receptor (wsMOR) can be derived in a very sensitive manner. Image courtesy of Prof. Jinglei Ping.
